# *In Vitro* Characterization of the Impact of Different Substrates on Metabolite Production, Energy Extraction and Composition of Gut Microbiota from Lean and Obese Subjects

**DOI:** 10.1371/journal.pone.0113864

**Published:** 2014-11-26

**Authors:** Marisol Aguirre, Daisy M. A. E. Jonkers, Freddy J. Troost, Guus Roeselers, Koen Venema

**Affiliations:** 1 Top Institute of Food & Nutrition, Wageningen, Gelderland, The Netherlands; 2 Department of Human Biology, Maastricht University, Maastricht, Limburg, The Netherlands; 3 Department of Microbiology & Systems Biology, The Netherlands Organization for Applied Scientific Research, Zeist, Utrecht, The Netherlands; Korea University, Republic Of Korea

## Abstract

The aim of this study was to investigate the effect of galacto-oligosaccharides, lactulose, apple fiber and sugar beet pectin on the composition and activity of human colonic microbiota of lean and obese healthy subjects using an *in vitro* model of the proximal colon: TIM-2. Substrate fermentation was assessed by measuring the production of short-chain and branched-chain fatty acids, lactate and ammonia and by studying the composition of the bacterial communities over time. The results suggest that energy harvest (in terms of metabolites) of lean and obese microbiotas is different and may depend on the fermentable substrate. For galacto-oligosaccharides and lactulose, the cumulative amount of short-chain fatty acids plus lactate produced in TIM-2 was lower in the fermentation experiments with the lean microbiota (123 and 155 mmol, respectively) compared to the obese (162 and 173 mmol, respectively). This was reversed for the pectin and the fiber. The absolute amount produced of short-chain fatty acids including lactate was higher after 72 h in the fermentation experiments with apple fiber-L (108 mmol) than with apple fiber-O (92 mmol). Sugar beet-L was also higher (130 mmol) compared to sugar beet-O (103 mmol). Galacto-oligosaccharides and lactulose boosted the balance of health-promoting over toxic metabolites produced by the microbiota from obese subjects. *Firmicutes* were more predominant in the inoculum prepared from feces of obese subjects compared to lean subjects. The average abundance at time zero was 92% and 74%, respectively. On the other hand, *Bacteroidetes* were more dominant in the microbiota prepared with homogenates from lean subjects with an average abundance of 22% compared with the microbiota prepared with homogenates from obese subjects (3.6%). This study brings evidence that different fermentable carbohydrates are fermented differently by lean and obese microbiotas, which contributes to the understanding of the role of diet and the microbiota in tackling obesity.

## Introduction

Overweight and obesity constitute two of the main risk factors for a wide range of chronic disorders, including diabetes (with a 44% increased risk in obese individuals), cardiovascular disease (23%), high blood pressure and cancer (7–41%) [1], [2].

A complex set of interactions have been unraveled between the colonic microbiota and humans, including a role in overweight and obesity. Studies with germ-free mice demonstrated that this group was leaner compared with mice with a normal gut microbiota, even when the latter were fed with a lower caloric diet. When microbiota was transplanted from normal into germ-free mice, an increase in body fat of 60% was observed [3]. In other studies, also mainly performed in rodents, obesity has been linked with increased levels of *Firmicutes* [4]. However, others have not been able to confirm this [5], [6], [7].

Given the importance of the intimate association between colonic microbiota and host, the microbiota has been acknowledged as a metabolic organ [8]. This metabolic function highly depends on the type of substrates available to be fermented by the microbiota in the gut [9]. The major fermentation processes in the colon are saccharolytic and proteolytic fermentation. The main metabolites formed during saccharolytic fermentation are short-chain fatty acids (SCFA: namely: acetate, propionate and butyrate) with some intermediate metabolites including succinate, acrylate, lactate, formate, ethanol, and gases such as H_2_, CH_4_ and CO_2_ [10]. On the other hand, proteolytic fermentation leads to the production of acetate, propionate, butyrate and branched-chain fatty acids (BCFA) including principally iso-butyric, iso-valeric and 2-methylbutyric acids [11]. Furthermore, it also results in the production of phenols, indoles, ammonia, and amines, which are recognized as potentially toxic metabolites to the host [9].

Acetate, propionate and butyrate have been of particular interest in many studies. Ferchaud-Roucher *et al.* [12] observed that acetate may interfere directly with lipid metabolism and compelling evidence has shown propionate to lower fatty acid content in liver and plasma. Furthermore, it has been found that propionate reduces food intake, exerts immunosuppressive actions and probably improves tissue insulin sensitivity [13], [14]. On the other hand, butyrate is metabolized by epithelial cells. It is responsible for 70% of their energy needs [15], [16] and acts as a signaling metabolite, affecting epithelial cell proliferation and differentiation [17], [18]. Although L- and D- lactate are found to be present in low concentrations in stools from healthy subjects, it has been observed that humans have the ability to metabolize both isomers to pyruvate via the L-lactate dehydrogenase (L-LDH) and D-2-hydroxy acid dehydrogenase [19]. Furthermore, significant amounts of lactate could be converted to SCFA, especially to butyrate, by some bacteria [20], [21]. For these reasons, its analysis was included in the present study.

As mentioned above, the concentration and ratio of SCFA produced in the gut depends, among others, on substrate availability and the composition of the intestinal microbiota [22]. Substrates for colonic fermentation include prebiotics. Prebiotics are defined as "non-digestible food ingredients that beneficially affect the host by selectively stimulating the growth and/or activity of one or a limited number of bacteria in the colon, thereby improving host health" [23]. Prebiotics also impact on the acidity of the lumen via the metabolic end-products (SCFA). By lowering the pH of the luminal content, prebiotics may exert a negative effect on harmful bacteria since acidification of the gut has been proposed as a mechanism that prevents the growth of pathogens [24]. The prebiotic property of influencing SCFA production in the gut may play an important role in the prevention and/or treatment of obesity.

With the increase of overweight and obesity worldwide and their devastating consequences on human health there is a growing interest to unravel the complex relationship between diet, gut microbiota, and host health. However, there is a lack of *in vitro* studies addressing such interaction experimenting with human gut microbiota. To our knowledge, one of the few studies that have investigated this role was performed by Sarbini *et al.* [25]. In this study, the fermentation of α-gluco-oligosaccharides and inulin produced similar effects on bacterial population and metabolic activity in both lean and obese microbiotas. Therefore, besides including microbiota from lean and obese subjects, this study incorporated the analysis of different fermentable prebiotics: galacto-oligosaccharides (GOS), lactulose, apple fiber and sugar beet pectin.

The reasons why the tested substrates were chosen are: i) the potential of GOS and lactulose to beneficially influence gut microbiota composition has not been widely studied [26]. Rowland and Tanaka [27] reported that GOS increased lactobacilli and bifidobacteria and decreased enterobacteria in rats colonized with human fecal microbiota. This was supported by Bouhnik *et al.* [28] and Maathuis *et al.* [29] who found that GOS administered to adults [28] and to the TIM-2 system [29] led to a significant increase in fecal amounts of bifidobacteria. Nevertheless some studies have not been able to replicate the bifidogenic impact of GOS [24]. Furthermore, little is known about the metabolic products from the fermentation of GOS and most of the time it has been studied in combination with fructo-oligosaccharides (FOS). ii) Lactulose has been found to selectively stimulate and decrease the growth of certain bacterial groups [23], contribute to colonization resistance [24], increase the absorption of calcium [30] and is fermentable by the gut microbiota [24]. The use of lactulose has been mainly in the pharmaceutical industry, and it has been commercialized in the last years as a drink additive or food [24]. Therefore, the impact of its consumption on the host microbiota is of particular interest. iii) Dietary fibers like pectins are important for nutritional companies because of their cheap production and their beneficial effects on reducing glucose absorption, hypocholesterolemic effects and delayed gastric emptying [31], [32]. Moreover, pectins may reduce the generation of ammonia [33] and the adhesion of pathogens to intestinal epithelial cells [34] in addition to their contribution in maintaining intestinal integrity [35]. Therefore, the influence of pectins on the intestinal microbiota composition was included here.

In the present work, the validated dynamic *in vitro* proximal colon model (TIM-2) developed by The Netherlands Organization for Applied Scientific Research (TNO) [36] was used to evaluate potential differences between the microbiotas originating from lean and obese individuals, and elucidate how energy is extracted from non-digestible carbohydrates or prebiotics in the form of SCFA products. The work contributes to the understanding whether dietary intervention helps to reduce obesity by suggesting that different fermentable carbohydrates are fermented differently by lean and obese microbiotas.

## Materials and Methods

### Test compounds and control

The compounds used as the carbohydrate source for the microbial fermentation in the *in vitro* system were GOS (97%, degree of polymerization (DP) 2–6, average DP 3, FrieslandCampina, Beilen, The Netherlands), lactulose (98%, Sigma-Aldrich, Zwijndrecht, The Netherlands), apple fiber (with a 60% content of total sugars 45% of them from glucose and 23% of uronic acid residues; CSM, Bingen, Germany) and sugar beet pectin (average degree of methylation of 53, and a 58% w w^−1^ content of uronic acids; GENU BETA pectin, CPKelco, Nijmegen, The Netherlands). A standard ileal efflux medium (SIEM; Tritium Microbiology, Veldhoven, The Netherlands) was used as a control and was modified from [37]. This medium contained the major non-digestible carbohydrates found in a normal western diet [29]. The preparation of starch (amylopectin and amylose) and non-starch polysaccharides (NSP; pectin, xylan and arabinogalactan) present in SIEM, have been used by other authors to simulate the dietary carbohydrate portion in fermentation studies [29], [38], [39]. In absence of mucin, Gibson *et al.*, [37] proposes this composite to provide a source of carbon during fermentation. As described by these authors, starch is included as a major polysaccharide due to evidence showing that this carbohydrate is highly abundant in the large intestine when compared with NSP. Furthermore, it is considered that resistant starch provides the largest proportion of energy to the colon [38]. Feed preparations were made containing approximately 7.5 g of fermentable carbohydrate per day, which constitutes 12% w w^−1^ of the total medium.

### Microbiota

Homogenates of human feces were made from subgroups of healthy volunteers who were selected depending on their body mass index (BMI). The group of participants were non-smokers and had not used antibiotics, prebiotics, probiotics or laxatives for at least 3 months prior to the donation.

Lean participants (n = 4) were recruited from a pool of volunteers at TNO Healthy Living (Zeist, The Netherlands). This group had an average BMI of 23.5±1.3 kg m^−2^.

Obese participants (n = 4) were recruited from a pool of volunteers at Maastricht University Medical Center (The Netherlands). The group had an average BMI of 33.5±2.6 kg m^−2^.

Fresh fecal samples were directly collected by all the donors in a closed box with an anaerobic strip (AnaeroGen, Oxoid, Cambridge, UK) inside. The collection of boxes for the study was carried out in a time frame of 5 h. As soon as a box was ready, it was stored in an anaerobic chamber (Bactron Anaerobic IV, Shel Lab, Cornelius, USA) to guarantee the best conditions for pooling. Donations from each category (lean or obese), were homogenized and mixed under strict anaerobic conditions to create a standardized microbiota stock. This allowed the performance of all experiments to start with the same microbial composition. Mixing was done with a Turrax (IKA Ultra turrax T25 digital, Staufen, Germany) with a physiological saline preparation/dialysate (content per litre: 2.5 g K_2_HPO_4_•3H_2_O, 4.5 g NaCl, 0.005 g FeSO_4_•7H_2_O, 0.5 g MgSO_4_•H_2_O, 0.45 g CaCl_2_•2H_2_O, 0.05 g ox bile and 0.4 g cysteine hydrochloride), and glycerol (14% w w^−1^) as cryoprotective agent. Aliquots of the fecal homogenates (±11% w w^−1^) were snap-frozen in liquid nitrogen (−196°C) and were stored in a freezer at −80°C [40]. Before being introduced into the system, the inoculum was thawed by 1 h immersion in a 37°C water bath.

Since this study demanded a high throughput experiments, it was decided to use a pool of microbiota as an inoculum for the different experiments. We have previously shown that pooling does not affect microbiota composition [41].

### TIM-2 experiments

**Figure 1:**
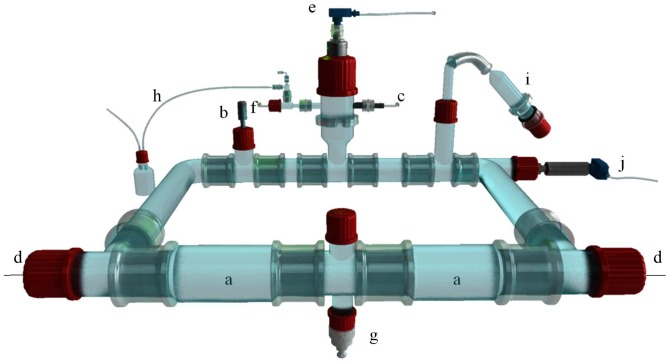
TIM-2 schematic, slightly modified from Maathuis *et al.*, [42]. a) peristaltic compartments with a hollow membrane inside, b) pH sensor, c) NaOH secretion, d) dialysate system, e) level sensor, f) gaseous N_2_ inlet, g) sampling port, h) gas outlet, i) test compound + SIEM feeding syringe, j) temperature sensor.

The TIM-2 system (Figure 1) has been described in detail before [42]. In brief, the units were flushed with N_2_ (Figure 1f) prior to the introduction of the inoculum, and throughout the remainder of the experiment. The system was maintained under this condition at 37°C using a temperature sensor (Figure 1j; Easytem R31, Endress + Hauser, Nesselwang, Germany) for 96 h with the pH kept at or above 5.8 by automatic titration with 2 M NaOH (Figure 1c). In order to remove water and fermentation products from the lumen a dialysate system (Figure 1d; described in the following section) consisting of a semi-permeable hollow membrane ran through the lumen. For all the experiments, the speed of the dialysis fluid was set at 1.5 ml min^−1^.

After 24 and 48 h of fermentation 25 ml of lumen sample was removed from the system to mimic the transit of chyme from the proximal to the distal colon [42].

The SIEM preparation mentioned above was gradually introduced into the system through a feeding syringe (Figure 1i) in a total volume of 40 ml in the adaptation period, while 180 ml of a modified SIEM-preparation (in which the standard carbohydrate source in the medium was replaced by the test compound) was used over the 72 h of the test period at a rate of 2.5 ml h^−1^. Luminal content was maintained at a level of approximately 120 ml in each unit by a level sensor (Figure 1e; liquiphant FTL20-0025, Endress + Hauser, Maulburg, Germany).

### Dialysate system

The dialysate fluid used in the system contained per litre: in addition to the dial preparation described under “microbiota” section, 1 ml of vitamin mixture containing per litre: 1 mg menadione, 2 mg biotin, 0.5 mg vitamin B_12_, 10 mg pantothenic acid, 5 mg nicotinamide, 5 mg *p*-aminobenzoic acid, and 4 mg thiamin (all from Tritium Microbiology).

### Fermentation

Each experimental unit of TIM-2 was filled to 120 ml with approximately 70 ml portion of fecal homogenate and 50 ml of dialysate. After inoculation, the microbiota was left to adapt (16 h) to the new environment. After this adaptation period, the microbiota was deprived from any carbohydrate source (starvation) for 4 h in order to completely use the carbohydrates in SIEM [43], which provides the best condition to assess the effects of the desired test compound without bias of the SIEM component from the adaptation phase. Minekus *et al.*, [36] explained that this starvation period was established when a lack of production of acids was observed in the system when the feeding line was turned off. This could be interpreted as the depletion of the fermentable carbohydrate.

**Figure 2:**
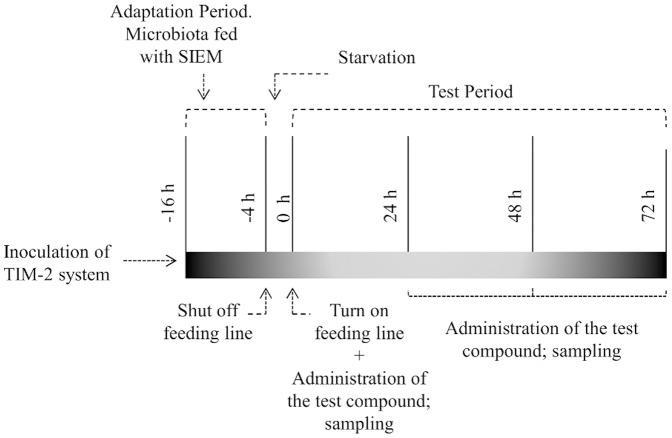
Study design.

After the starvation period, an initial lumen and dialysate portion were taken for later analysis (t = 0) and the microbiota was immediately fed with preparations containing the substrates under study (Figure 2). With samples from lumen and dialysate taken every 24 h of the experiment the production of SCFA, BCFA, lactate and ammonia was calculated. The concentration of metabolites at t = 0 was artificially set to zero. The microbial composition was measured at t = 0 and t = 72 in the luminal samples.

### Analytical methods

SCFA (acetate, propionate and butyrate), BCFA (iso-butyrate and iso-valerate), lactate and ammonia analyses were performed as described by Van Nuenen *et al.* [44]. Calibration lines were obtained by injecting known quantities of standard solutions, and amounts in the samples were calculated based on the calibration lines. Lactate and ammonia analyses were performed by Bio-aNAlytiX (Mook, The Netherlands).

### Energy extraction

Energy extraction in the form of SCFA was calculated using the following kJ mol^−1^ values for acetate, propionate, butyrate and lactate respectively: 874, 1536, 2192, and 1364 [45], [46].

### Molecular techniques

DNA from the luminal samples was isolated using the AGOWA mag Mini kit (DNA Isolation Kit, AGOWA, Berlin, Germany), according to the manufacturer's instructions.

Generation of the PCR amplicon library was performed by amplification of V5–V7 hypervariable region of the small subunit ribosomal DNA gene (16S rDNA). Amplification was performed using the forward primer 785F (5′GGATTAGATACCCBRGTAGTC-3′) and reverse primer 1175R (‘5- ACGTCRTCCCCDCCTTCCTC-3). The primers were fitted with the Roche 454 (Branford, CT, USA) Adapter A (forward primer) and B (reverse primer), fused to the 5′ end of the 16S rDNA bacterial primer sequences. The forward primer also included a unique nucleotide barcode. The amplification mix contained 2 units of Pfu Ultra II Fusion HS DNA polymerase (Stratagene, La Jolla, CA, USA) and 1x Pfu Ultra II reaction buffer (Stratagene), 200 µM dNTP PurePeak DNA polymerase Mix (Pierce Nucleic Acid Technologies, Milwaukee, WI, USA), and 0.2 µM of each primer.

After an initial denaturation (94°C; 2min), 30 cycles were performed that consisted of denaturation (94°C; 30sec), annealing (50°C; 40sec), and extension (72°C; 80 sec). Samples with DNA recovery of equal or less than 10 pg µl^−1^ of DNA were cycled 35 times using the same protocol.

Amplicons were size checked and quantified by gel electrophoresis and Quant-iT Picogreen dsDNA Assay (Invitrogen, Carlsbad, CA, USA) on the Tecan Infinite M200 (Tecan Group Ltd, Männedorf, Switzerland). Amplicons of the individual samples were equimolar pooled and purified from agarose gel by means of QIAquick Gel Extraction Kit Protocol (Qiagen, Hilden, Germany).

The library was sequenced unidirectionally in the forward direction (A-adaptor) in one run in the 454 GS-flx-Titanium Sequencer (Roche) by Keygene (Wageningen, The Netherlands).

### Sequence processing and analyses

FASTA-formatted sequences and corresponding quality scores were extracted from the .sff data file generated by the GS-FLX-Titatium sequencer using the GS Amplicon software package (Roche).

All data extraction, pre-processing, analysis of operational taxonomic units (OTUs), and classifications were performed using modules implemented in the Mothur v. 1.20.0. software platform [47] as in Roeselers *et al.* [48].

In brief, unique barcodes were used to sort sequences by sample of origin. Subsequently, barcodes, primer sequences and low quality data (containing ambiguous base calls (N) in the sequence, more than 8 homopolymers anywhere in the sequence, shorter than 50 nt after trimming with a window average below 35 or a length >500 or <200 bp) were removed. The data set was simplified by using the “unique.seqs” command to generate a non-redundant (unique) set of sequences. Sequences were ‘denoised’ using the “pre.cluster” command [49]. Unique sequences were aligned using the “align.seqs” command and an adaptation of the Bacterial SILVA SEED database as a template [50]. In order to ensure that comparable regions of the 16S rDNA gene were analyzed across all reads, sequences that started before the 2.5-percentile or ended after the 97.5-percentile in the alignment were filtered.

A total of 149220 potentially chimeric sequences were detected and removed using the “chimera.uchime” command [51]. High quality aligned sequences were classified by using the RDP-II naïve Bayesian Classifier [52] using a 60% confidence threshold. Aligned sequences were also clustered into operational taxonomic units (OTUs; defined by 97% similarity) and were calculated by the average linkage clustering method. Unclassified sequences were also grouped in OTUs and were represented with a random number to distinguish them from other unclassified OTUs found within the same phyla. Community profiles were compared using the weighted Unifrac metric [53].

### Data presentation

The experiments were performed in duplicate (n = 2) per substrate, except for control (n = 3). To avoid unnecessary repetition, this is not indicated further in the text or graphs in the results section. Results are displayed as average of these duplicates/triplicates. Since the sample size is small no statistical analysis was performed. For simplicity of reading, substrates in the following sections are tagged with the letter L or O (e.g. substrate-L, substrate-O) in order to refer to the fermentation experiments using the inoculum from lean (-L) or obese (-O) subjects.

### Ethical approval

Studies using fecal donations from healthy volunteers do not require medical ethical committee approval in The Netherlands since they are considered as non-invasive. Nevertheless, all participants provided informed consent.

## Results

### Metabolites

**Figure 3:**
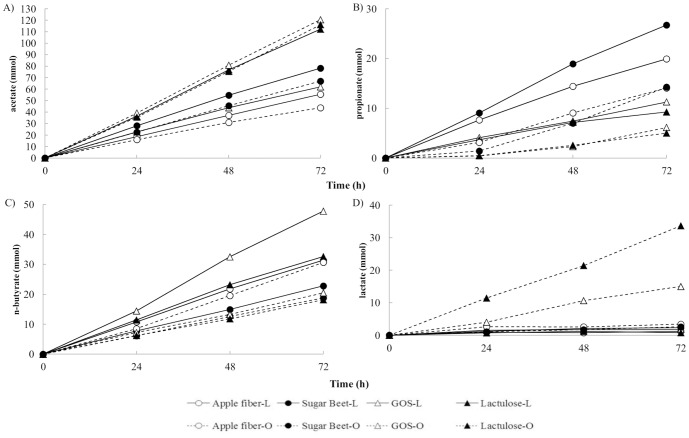
Cumulative production of SCFA. A) acetate, B) propionate, C) *n*-butyrate and D) lactate produced during 72 h fermentation of apple fiber, sugar beet pectin, GOS and lactulose using microbiota from lean or obese subjects.

The cumulative amount of SCFA including lactate produced in TIM-2 (Figure 3) was lower after 72 h in the fermentation experiments with GOS-L (123 mmol) compared to GOS-O (162 mmol). Also lactulose-L resulted in lower production than lactulose-O with values of 155 mmol and 173 mmol, respectively. In contrast to the results obtained for GOS and lactulose, the fermentation of apple fiber and sugar beet pectin with microbiota from lean subjects exhibited a higher cumulative production of SCFA compared with microbiota from obese subjects. With respect to apple fiber-L, the amounts were higher (108 mmol) than with apple fiber-O (92 mmol). Sugar beet-O (103 mmol) was lower than sugar beet-L (130 mmol). The amount for the SIEM control-L (125 mmol) and that for control-O (124 mmol) were very similar. The averaged molar ratios (% of total SCFA) are shown in Figure S1. In general, acetate was the main product in the different fermentations followed by *n*-butyrate (with the exception of lactulose-O and sugar beet-L in which it was exceeded by lactate and propionate, respectively) and propionate. Specifically in the case of lactate, it can be observed that its production was higher in GOS-O and lactulose-O experiments when compared to the other substrates (Figure 3 and Figure S1).

Fermentations of the different substrates using microbiota from lean subjects in TIM-2 resulted in higher amounts of BCFA and ammonia compared to fermentations using the obese inocula (Table 1). An approximately 4.5 and ∼10.5 fold increase of BCFA were observed for GOS-L and lactulose-L compared to the O-microbiota, whilst both presented 1.1 fold increase in ammonia. Interestingly, for GOS and lactulose, the amounts of BCFA and ammonia were lower than their respective controls for both L and O inocula. The cumulative BCFA produced by the lean microbiota in the incubations with apple fiber and sugar beet pectin were also higher: ∼1.7 fold for apple fiber and ∼2.9 fold for sugar beet pectin. The ammonia increase in both substrates was ∼1.1 and ∼1.3 fold, respectively.

**Table 1:** Cumulative production of BCFA and ammonia after 72 h of addition of apple fiber, sugar beet pectin, GOS or lactulose.

	Total amount (mmol) after 3 day experiments	
Substrate	*i*-butyrate + *i*-valerate	Ammonia
Apple fiber-L	4.5	65
Sugar beet-L	4.1	74
GOS-L	1.8	44
Lactulose-L	2.1	36
Apple fiber-O	2.6	59
Sugar beet-O	1.4	59
GOS-O	0.4	39
Lactulose-O	0.2	33
Control-L	3.9	74
Control-O	0.7	52

### Energy extraction

**Figure 4:**
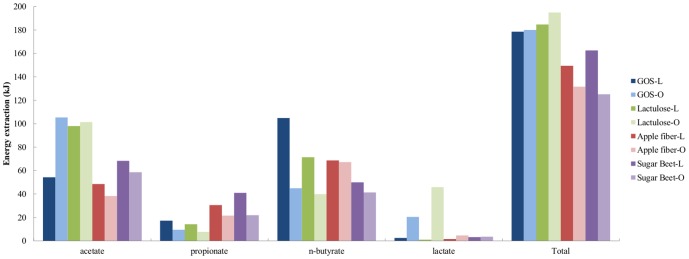
Energy extraction. Values obtained after 72 h fermentation of GOS, lactulose, apple fiber and sugar beet using microbiota from lean or obese subjects. Total energy extraction for control-L and -O were 176 and 162 kJ, respectively. The calculation was performed by multiplying the SCFA and lactate produced by their corresponding kJ mol^−1^ value as explained in the materials and methods section.

Three day fermentation of GOS provided an energy value in the form of SCFA and lactate of 180 kJ in experiments with both L and O microbiota, despite their total SCFA concentrations being different. The amount of energy extracted is higher as their respective controls (176 and 162 kJ, respectively for control-L and -O). Fermentation of lactulose-L extracted 185 kJ in the form of SCFA and lactate, whereas for lactulose-O this was 195 kJ (Figure 4). These results suggest that GOS and lactulose had a greater energy value per gram carbohydrate when compared to control.

Apple fiber had an energy value of 149 and 132 kJ after 72 h of fermentation, for experiments with lean and obese inocula, respectively. That is less compared to their respective controls. Fermentation of sugar beet-L extracted 162 kJ of energy whereas for sugar beet-O this was 125 kJ. These results suggest that apple fiber and sugar beet pectin in both types of fermentations had a lower energy value per gram when compared to control, and that contrary to expectations, the lean microbiota extracted more energy from these two substrates than the obese microbiota.

### Phylogenetic analysis of the microbiota at t = 0

**Figure 5:**
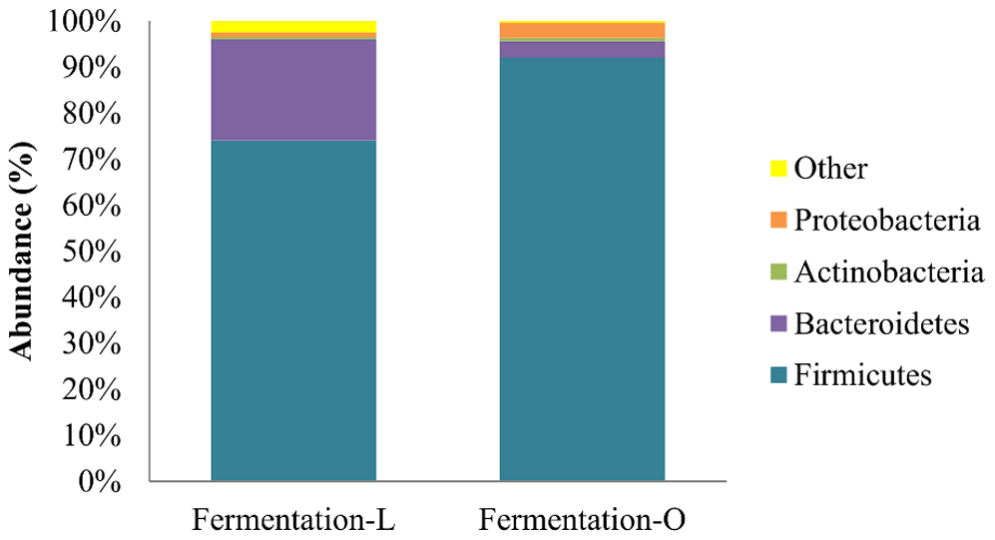
Abundance (%) of *Firmicutes, Bacteroidetes, Actinobacteria, Proteobacteria* and others. Phyla were analyzed in the fermentations with microbiota from lean (L) or obese (O) subjects at time zero. Data from all runs (fermentation-L n = 11; fermentation-O n = 10) were taken. The category presented as “Other” includes the sum of *Verrucomicrobia, Lentisphaerae, Tenericutes* and unclassified bacteria which constituted a low abundance in the samples.

After the adaptation period in the TIM-2 system, *Firmicutes* were more predominant in the microbiota prepared with the homogenates of obese subjects compared to that prepared with the homogenates from lean subjects (Figure 5). The average abundance at time zero was 92±3.3% (n = 10) and 74±1.0% (n = 11), respectively. On the other hand, *Bacteroidetes* were more dominant in the microbiota prepared with homogenates from lean subjects with an average abundance of 22±0.9% (n = 11) compared with the microbiota prepared with homogenates from obese subjects (3.6±1.9%; n = 10).

A low proportion of *Actinobacteria* and *Proteobacteria* was observed in the microbiota from the obese (0.7±0.3% and 3.3±1.1%) and lean individuals (0.5±0.2% and 1.0±0.3%).

### Changes observed in the microbiota after the experiments in TIM-2

A phyla and genus level analysis was performed using the sequencing data in which changes in bacterial abundance were measured by considering the changes in the two controls (-L and –O) as the baseline. The ratio between the shift in the composition of the microbiota in the experiments using different substrates and the shift of the microbiota fed with the control carbohydrate was calculated, presented as a “fold increase or decrease”. Several groups of bacteria showed changes in their relative abundance (Table 2), as detailed below.

**Table 2:** Relative change of bacterial genera after 72 h of fermentation experiments in TIM-2^1^.

Phyla	Genus	Apple fiber-L	Sugar beet-L	GOS-L	Lactulose-L	Apple fiber-O	Sugar Beet-O	GOS-O	Lactulose-O
	Catenibacterium	215.0		0.5	15.0				
	Anaerostipes	46.4		0.6	22.7	27.2		6.2	29.7
	Enterococcus	28.7				2.1	0.4	5.0	7.6
	Clostridium IV	19.3	1.4	0.1	0.2				
	Clostridium XlVb	17.80	20.6	0.03	11.9				
	OTU 5111	6.1	1.1	0.04					
	Blautia	5.7	1.5	2.0	4.8	2.7	0.4	29.7	50.8
	Acetivibrio	5.2	3.6						
	Coprococcus	3.0	1.1	0.1	1.8	16.0	2.3	1.1	6.6
	Roseburia	1.8	0.1		1.5		0.2		
	Erysipelotrichaceae incertae sedis	1.60	0.4	0.0	16.8				
	OTU 2128	1.6	0.3	0.1	0.3	1.6	0.6	3.8	0.6
	OTU 1291	1.5	1.0	0.2	0.5	6.4	6.9	27.1	2.0
	Clostridium XlVa	1.4	0.4	4.9	0.3				
	Faecalibacterium	1.2	6.0	0.5	0.6	1.4	8.1	22.2	13.5
***Firmicutes***	Dorea	1.1	0.2	0.1	0.7	46.1	6.2	38.5	48.2
	OTU 2620	0.84	0.8	0.01	0.1		9.9	6.4	8.8
	Ruminococcus	0.56							
	Subdoligranulum	0.47	0.6	0.01	0.1	0.03	0.8		0.4
	OTU 2832	0.31		0.04	0.1				
	Oscillibacter	0.3	0.7	0.02	0.3				
	Lachnospiracea incertae sedis	0.10	0.1	1.4	0.2	22.96	4.0	0.4	0.3
	Dialister	0.04	0.4	0.04	0.1				
	Lactobacillus					8.5	0.2	0.9	4.9
	Lactococcus						0.2	13.4	
	Streptococcus			33.1		4.8	0.2	2.7	7.6
	Clostridium sensu stricto					1.2	0.3	1.1	1.4
	Anaerococcus								0.8
	Anaerovorax		0.6		0.2				
	Clostridium XI								5.7
	Butyricicoccus					0.1	0.2	1.0	0.2
	Allisonella					3.6	4.6	2.8	9.3
***Bacteroidetes***	Parabacteroides	6.5	2.8	2.1	2.0	0.3	6.1	0.4	0.2
	Bacteroides	4.3	21.6	9.1	1.2	1.9	2.5	0.2	0.1
	OTU 2171	3.81	0.6	7.2	0.3				
	OTU 2145	1.8	1.3	0.3	0.6				
	Alistipes	1.45	0.6	0.05	3.8				
	Xylanibacter	1.1	0.9	0.1					
	Prevotella	0.6	0.8	0.01	0.01				
	Barnesiella	0.1			0.2				
***Actinobacteria***	Bifidobacterium		12.5	600.1	4338.1	0.04	0.3	99.5	890.1
	Collinsella			0.2	0.3				
	OTU 4114						2.0	2.8	5.5
***Proteobacteria***	Parasutterella	10.7	9.9	0.5	27.6				
	Gemmiger	0.9	1.4		0.2				
	Sutterella			0.2					
	OTU 1144					1.1	0.32	21	8.9
**Unclassified**	OTU 1111	0.5	0.8	0.0	0.1	0.04	0.02	0.01	0.01

1 The ratio between a sampling time point and t_−16_ was calculated (e.g., t_72_/t_−16_). The ratio for this value and the pool was then determined to obtain fold changes. A value equal to 1 indicates no change; a value of >1 indicates an increase; and a value of <1 indicates a decrease of the respective microbial genera. Sequences of the 16S rDNA gene that are not fully available in the database but represent a high similarity in the genus tagged are presented as unclassified groups and are listed in the table as OTU###, with ### being a number.

#### GOS and lactulose

The dynamics for *Firmicutes* and *Bacteroidetes* was different in all the fermentations with GOS and lactulose. GOS-L presented an increase in *Firmicutes* with the biggest raise in *Streptococcus* with a fold increase of 33, whereas GOS-O exhibited the biggest increase in *Dorea spp.* (39 fold). The fermentation with lactulose-L stimulated the growth of *Anaerostipes spp.* (23 fold) whilst lactulose-O exhibited a remarkable increase of *Blautia spp.* (51 fold).

A modest increase in *Bacteroidetes* was observed in the experiments with the inoculum prepared from lean subjects. GOS-L fermentation led to an increase in *Bacteroides spp.* (9 fold) whereas lactulose-L exhibited a moderate increase in *Alistipes spp.* (4 fold). Both GOS-L and lactulose-L presented a 2 fold increase in *Parabacteroides spp.* In the case of the experiments with the inoculum from obese subjects a general decrease of *Bacteroidetes* with respect to control was observed for both GOS and lactulose. The bacterial group corresponding to *Parabacteroides spp.* presented the lowest values: 0.38 fold (or 2.6 fold decrease) and 0.21 fold (4.8 fold decrease) for GOS and lactulose, respectively.

An outstanding growth in the group of *Actinobacteria* was found after 3 days of experiments with both GOS and lactulose as shown in Table 2. Lactulose-L presented a robust increase (4300 fold) compared with control followed by lactulose-O (890 fold), GOS-L (600 fold) and GOS-O (100 fold).

In the case of *Proteobacteria* there was a substantial increase in *Parasutterella spp.* in lactulose-L (28 fold). Although reported as an unclassified OTU (1144) by the sequencing analysis, a large increase was also observed for GOS-O (21 fold) and lactulose-O (9 fold) in this genus. A decrease of 0.52 fold of *Proteobacteria* was found in GOS-L in the specific case of *Parasutterella spp.*

#### Apple fiber and sugar beet pectin

OTUs from *Firmicutes* which were observed to increase the most for each fermentation that was carried out with apple fiber and sugar beet pectin corresponded to *Catenibacterium spp*. (215 fold; apple fiber-L), *Dorea spp.* (46 fold; apple fiber-O), *Clostridium XIVb* (21 fold; sugar beet pectin-L) and *Faecalibacterium* (8 fold; sugar beet pectin-O).

For *Bacteroidetes*, the fermentations with apple fiber-L and sugar beet pectin-O presented the same increase of *Parabacteroides spp.* (each 6 fold). *Bacteroides spp.* was found to increase (22 fold) in sugar beet pectin-L.

The proliferation of microorganisms from the group of *Actinobacteria* was not stimulated in both fermentations (-L and –O) with apple fiber. On the other hand, experiments with sugar beet pectin-L were found to stimulate the growth of *Bifidobacterium* (12 fold) whilst a moderate increase (2 fold) of unclassified *Actinobacteria* (OTU 4114) was observed in sugar beet pectin-O.

On the other hand, increases in some *Proteobacteria* groups were observed in experiments with the lean microbiota such as the case apple fiber-L and sugar beet pectin-L which showed a similar increase in *Parasutterella spp.* (11 and 10 fold, respectively).

## Discussion

The differences between human microbiotas from lean and obese individuals in terms of composition and activity, and their contribution to obesity through energy extraction in the form of SCFA and lactate was studied *in vitro* by fermenting several non-digestible, but fermentable carbohydrates. The golden standard to test this would be in a clinical trial. However, such trials are highly expensive and present certain limitations in design due to throughput and ethical constrains. Therefore, before going to sometimes rather invasive human intervention studies, *in vitro* models closely mimicking the microbial metabolism in the human intestine can be used to get further insight in the complex fermentation processes mediated by the gut microbiota.

TIM-2 is such an *in vitro* model that closely mimics the fermentation by the microbiota in the human large intestine by allowing the growth of a highly complex, stable and dense (∼10^11^ colony forming units/ml) active microbiota [43], [54]. The variations in the system are small, due to the fact that the experiments are computer-controlled and a standardized microbiota is used for each set of experiments which is derived from host subjects of interest [6]. In addition, the system has been validated analyzing i) the composition, ii) the enzymatic activity and iii) the production and concentration of SCFA of the microbiota used for the experiments [36], based on data from sudden-death individuals. Overall, the model provides the opportunity to study the human microbial ecosystem and the metabolism of test substrates under standardized conditions [40], [42]. However, a limitation of the study set up may be the fact that a pool of microbiota rather than the microbiota from a single individual was used in the experiments. This is criticized mainly due to lack of stability of the mixed inoculum due to bacterial interactions. Nevertheless, this was done with the aim of working with i) a standardized microbiota that can be used for multiple (up to 100) different experiments, and ii) a more diverse population of bacteria and thus the use of a more representative microbiota for the whole population.

To our knowledge no comparative experiments have been performed using a single or mixed fecal inoculum. However, in a recent study performed by us in TIM-2, it was possible to demonstrate that the use of a pool of microbiota for *in vitro* studies does not result in a bacterial community with an aberrant profile and activity compared to that normally obtained from single donors [41]. In other studies using TIM-2 (pooled microbiota) as well as by the Ghent University in their Simulator of the Human Microbial Ecosystem (SHIME) (single fecal inoculum) with arabinoxylan very similar metabolic activity of the microbiota was observed [55]. In addition, previous data using the individual inocula from 10 donors has shown that the microbial activity of the individual donors was extremely similar, despite differences in microbiota composition [56], corroborating the hypothesis that there is substantial functional overlap in the microbiota of different individuals and that a standardized mixture of gut microbiota can be used for these type of experiments.

### Metabolites produced

Based on experiments with ^13^C-labeled substrates, it was shown that SCFA make up >95% of the metabolites produced by the microbiota when fermenting a carbohydrate substrate [43]. Therefore, we focused on these metabolites when determining energy extraction. The fermentation of complex carbohydrates by bacteria has been considered as the major quantitative source of SCFA in the colon. Nevertheless, it is important to consider that breakdown of peptides and proteins do also contribute to the formation of SCFA as seen *in vivo* and *in vitro* [57]. Therefore, the possibility exists that some of the observed SCFA produced in the different experiments could also come from the fermentation of proteins. However, only the carbohydrate part of the medium was changed between the different variables tested. Consequently, the levels of protein in the diet used in the experiment and their conversion to SCFA are expected to be fairly similar for every experiment as well (including controls). Hence, the contribution of proteins to the energy extracted by the microbiota in terms of SCFA is also believed to be fairly similar regardless of the type of the test compound studied.

In the current experiments, low levels of lactate were found for both GOS-L and lactulose-L. On the other hand, high proportions of butyrate found in both fermentations were observed (Figure S1) likely as a result of conversion of lactate into butyrate [58]. Butyrate is considered a healthy microbial metabolite [59]. From this, it is proposed that GOS and lactulose in lean people could boost a healthier colonic environment through conversion of lactate into butyrate.

In fermentations with GOS-O and lactulose-O there is a slight increase in total SCFA produced from both test compounds (1.3 fold for GOS-O and 1.1 fold for lactulose-O) compared to lean. It is important to highlight the fact that an increase in lactate was observed in these experiments as well which can be interpreted that GOS and lactulose are fermented faster in obese than in lean subjects, since lactate normally only accumulates when fermentation is fast [44].

Apple fiber and sugar beet pectin were more poorly fermented in all the experiments when compared with control, GOS and lactulose, as observed by the total production of SCFA and energy extraction. It can be observed that these substrates were not equally used by the microbiota from lean and obese subjects as evidenced by the nature of SCFA produced from the fermentation of these compounds. In contrast to the results obtained for GOS and lactulose, the fermentation of pectins with microbiota from lean subjects exhibited a higher cumulative production of SCFA compared with microbiota from obese subjects (1.2 fold for apple fiber-L and 1.3 fold for sugar beet pectin-L), suggesting a potential relative reduction of the presence of pectate lyases and other enzymes involved in pectin fermentation in the obese microbiota.

It is proposed that the sugar composition from the different test compounds could influence the type of the SCFA produced. Salvador *et al.* [60] found a correlation between high concentrations of acetate and uronic acid content from sugar beet in fermentations with fresh feces from healthy volunteers in an *in vitro* batch system. The results from the present study are in agreement with Salvador *et al*. Fermentations with sugar beet pectin in our experiments exhibited a higher amount of acetate when compared to apple fiber (1.4 fold in fermentation-L and 1.5 fold in fermentation-O) (Figure 3). The sugar beet pectin contained 80 mol% of uronic acid whereas apple fiber only had 23 mol%. Furthermore, Salvador *et al.* also demonstrated that butyrate production from fiber fermentation could be positively associated with the content of xylose. Taking into account that apple fiber contained 6 mol% of xylose whilst sugar beet lacked xylose, the present study is in accordance with the findings from these authors, as we show here that apple fiber exhibited a higher butyrate production when compared to sugar beet pectin (1.3 fold in fermentation-L and 1.6 in fermentation-O).

In the experiments with microbiota-O, the addition of GOS and lactulose suppressed the formation of putrefactive metabolites compared to control. The results obtained demonstrate the potential of GOS and lactulose to reduce toxic metabolic products by repressing protein fermentation in obese subjects, providing good indication of their prebiotic effect.

### Energy extraction

A higher production of SCFA by the microbiota will consequently lead to a higher energy input to the host, as 95% of the SCFA produced by the microbiota are absorbed, and only 5% is excreted in feces [61]. Yet, it is important to realize that each SCFA contributes differently to the energy extracted from fermentable carbohydrates. Butyrate contains more energy than acetate and propionate (2192, 874 and 1536 kJ mol^−1^, respectively) [6]. In this study, the energy extracted from GOS and lactulose for both types of fermentation was similar. However, it was observed that *n*-butyrate was higher in the fermentations with the lean inocula and therefore, the caloric input through *n*-butyrate was increased. Both apple fiber and sugar beet pectin exhibited more energy input in experiments with the lean inocula. The obese microbiota fermenting the fiber and the pectin produced less energy from acetate and propionate compared with the lean one. The energy input from propionate was higher in sugar beet-L when compared to the other fermentations (Figure 4). It could be that, as obese subjects have a different type of diet (low in complex carbohydrates), this results in a non-adapted microbiota, not fully capable of degrading pectins. Important to consider as well is the pH of the system (pH 5.8, simulating the proximal colon) which might also have inhibited the activity of pectate lyases. Further experiments are carried out in order to confirm these hypotheses.

It is believed that the additional energy input to the host from its microbiota could be used for glucose and *de novo* hepatic triglyceride synthesis [62]. A slight increase of just 1% in the microbial metabolic activity may increase the input of 20 kcal day^−1^ to the host (based on a diet of 2000 kcal day^−1^) which could lead to a weight gain of approximately 1 kg per year [62]. For this reason, the impact of the activity of gut bacteria on obesity in humans should not be underestimated.

### Phylogenetic analysis of the microbiota at t = 0

The phylogenetic analysis of the inoculum prepared with microbiota-L and microbiota-O at time zero (after the adaptation period) demonstrates that both individual groups cluster closely together depending on the source of the inoculum (Figure S2). This demonstrates the difference between these two types of inocula at the starting point of the experiments. Furthermore, this shows the high reproducibility of the *in vitro* model.

When calculated, the ratio of *Firmicutes/Bacteroidetes* in the starting microbiota prepared with donations from obese subjects at time zero was found to be higher (87±168) compared to the starting microbiota prepared with donations from lean subjects (4.5±4.2). In studies, mainly performed in rodents, obesity has been linked with reduced levels of *Bacteroidetes* [5], [6], [7]. In fact, in a study conducted in obese mice resulted in more efficient energy harvesting compared to lean counterparts, this was associated with a higher ratio of *Firmicutes/Bacteroidetes* in these animals [4], which corroborates our observations. Although it is hypothesized that this increased ratio might either promote fat storage or lead to a different energy uptake/storage in the body, the exact mechanisms are not known. In contrast, other studies such as the one conducted by Duncan *et al.* [5] did not find any evidence about the function of proportions of *Bacteroidetes* and *Firmicutes* in human obesity.

### Changes observed in the microbiota after the experiments in TIM-2

*Bifidobacterium* from both lean and obese subjects were clearly stimulated by GOS and lactulose during the 3 day fermentation period, as the increase in this group of microorganisms was much higher compared with the other genera. The metabolic activity of bifidobacteria has been found to be beneficial to the host, *e.g*., it has shown to inhibit the colonisation of pathogenic microorganisms, and have anti-carcinogenic effects [63]. Important to realize is that not all strains of bifidobacteria have probiotic effects. It has been found that some bacteria from this genus are implicated in mucin degradation, whilst others are recognized as β-glucosidase producers hypothesized to be involved in colon cancer promotion [64]. A robust increase of *Bifidobacterium* spp. likely causes a higher production of acetate and lactate [29], which is indeed evidenced by the higher ratios of these SCFA in those experiments where bifidobacteria are increased.

The markedly increased abundance of *Catenibacterium spp* in apple fiber-L could explain why the cumulative SCFA ratios were higher compared with sugar beet-L. According to Kageyama and Yoshimi [65], the major end products from the fermentation of carbohydrates by *Catenibacterium spp.* are acetate, butyrate and lactate. Thus, this demonstrates the high yield in the energy extraction from the fermented apple fiber in lean subjects.

The results also evidence the potential benefit of apple fiber and sugar beet pectin for obese and lean individuals, respectively. On the one hand, it is presumed that *Dorea* spp. (46 fold increase in apple fiber-O) are related in blunting inflammatory processes [66], [67]. On the other hand, *Clostridium* cluster XIV spp. (21 fold increase in sugar beet-L) may lead to a reduced circulation of inflammatory markers and body weight [68].

The prebiotic effect of sugar beet pectin is also demonstrated by the stimulation of the growth of *Faecalibacterium* spp. *F. prausnitzii* is considered as an anti-inflammatory species which stimulates the production of butyrate [69]. As Furet *et al.* [70] found low abundance of this bacteria in obese subjects, the potential beneficial effect of sugar beet pectin for these subjects is highlighted by the observed increase in *Faecalibacterium*. More studies at the level of bacterial species and the measurement of other markers including especially inflammatory ones are recommended in order to assess the effect of these two substrates in the colonic homeostasis in humans.

The results from the present study suggest differences in the extraction of energy in the form of SCFA as well as dynamic changes in the composition of the gut bacteria which may be strongly associated with the substrate provided to the microbiota and its origin (*i.e*., lean or obese individuals).

## Supporting Information

Figure S1 Averaged molar ratios for acetate, propionate, *n*-butyrate and lactate (% of total SCFA).
(TIF)

Figure S2 (A) Weighted UniFrac tree of 16S rDNA pyrosequences. Spanning of the V5–V7 hypervariable regions derived from the different TIM microbiotas (green for L; red for O). Data shown corresponds to a single run after the adaptation period without the addition of the test compound. Scale bars indicate distance between the samples in UniFrac units. (B) The relative abundance of bacterial orders observed in these data sets is represented in a heatmap, showing those bacterial groups that contribute largely to the difference between the two clusters.
(TIF)
