# Supplementary figures and images for: In Vitro Characterization of the Impact of Different Substrates on Metabolite Production, Energy Extraction and Composition of Gut Microbiota from Lean and Obese Subjects
Source: PLoS One. 2014 Nov 26;9(11):e113864. doi: 10.1371/journal.pone.0113864 (PMC4245234; doi:10.1371/journal.pone.0113864)

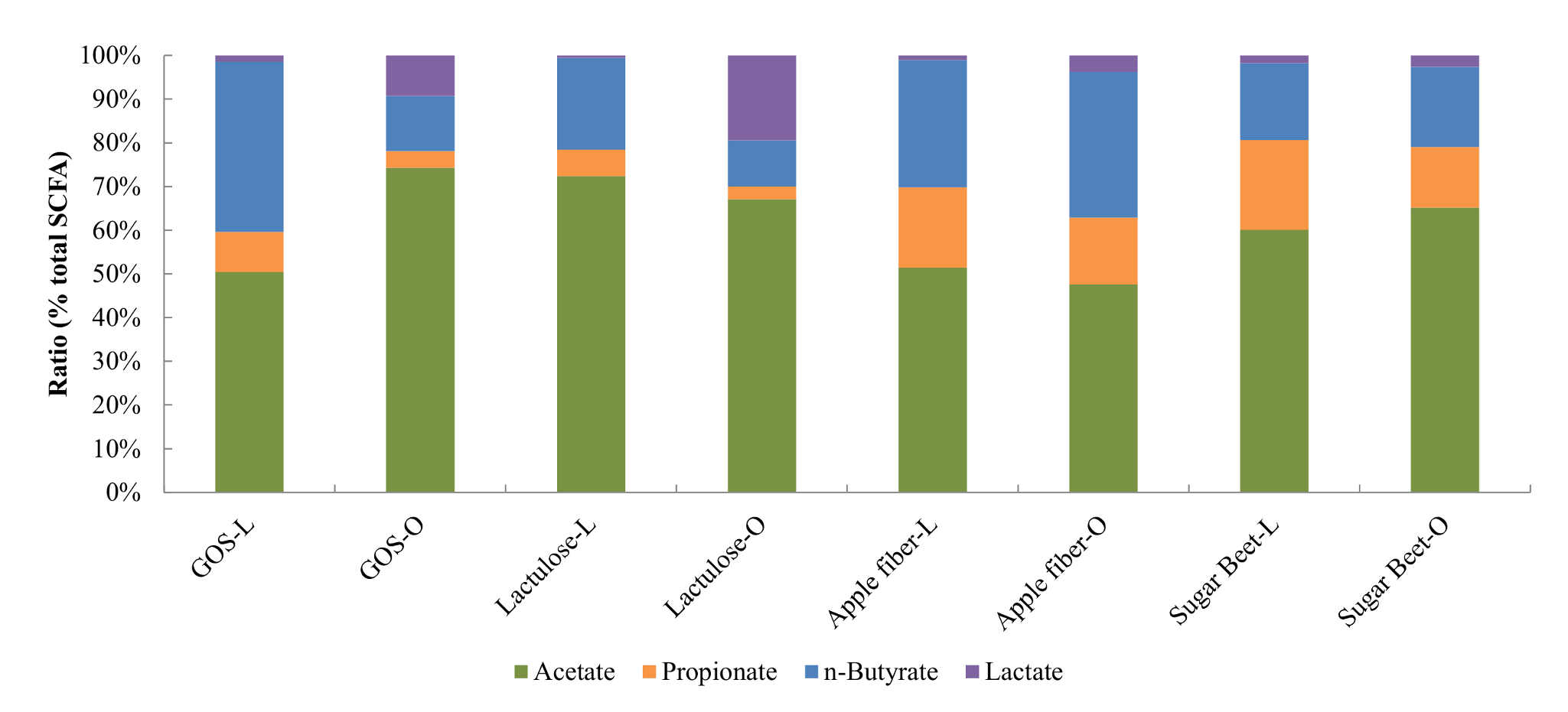

Supplement: Figure S1 — Averaged molar ratios for acetate, propionate, n-butyrate and lactate (% of total SCFA). (TIF) [file pone.0113864.s001.tif]

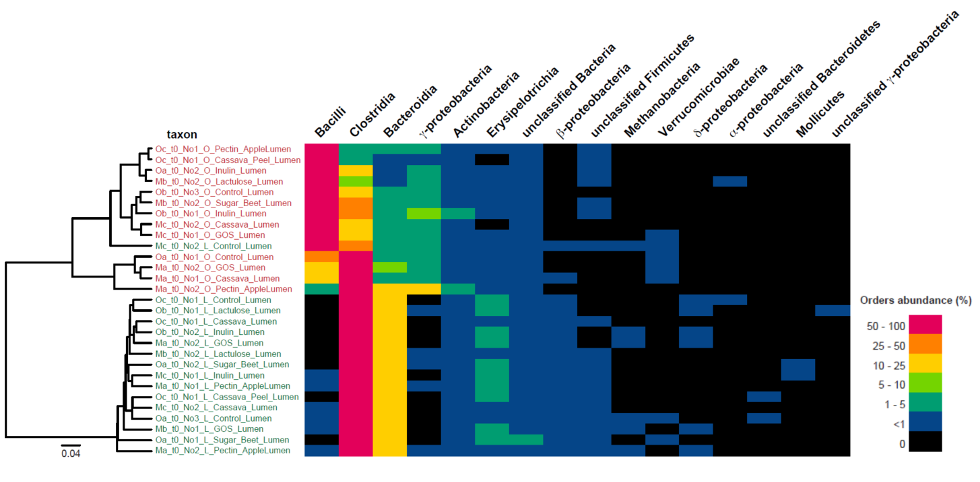

Supplement: Figure S2 — (A) Weighted UniFrac tree of 16S rDNA pyrosequences. Spanning of the V5–V7 hypervariable regions derived from the different TIM microbiotas (green for L; red for O). Data shown corresponds to a single run after the adaptation period without the addition of the test compound. Scale bars indicate distance between the samples in UniFrac units. (B) The relative abundance of bacterial orders observed in these data sets is represented in a heatmap, showing those bacterial groups that contribute largely to the difference between the two clusters. (TIF) [file pone.0113864.s002.tif]
